# Enhanced SOC estimation of lithium ion batteries with RealTime data using machine learning algorithms

**DOI:** 10.1038/s41598-024-66997-9

**Published:** 2024-07-11

**Authors:** Obuli Pranav D., Preethem S. Babu, Indragandhi V., Ashok B., Vedhanayaki S., Kavitha C.

**Affiliations:** 1 School of Electrical Engineering, VIT, Vellore, India; 2 School of Mechanical Engineering, VIT, Vellore, India; 3 Department of Electronics and Communication Engineering, Sreenivasa Institute of Technology and Management Studies, Chittoor, India

**Keywords:** State of charge, Machine learning, Electric vehicle, Lithium-ion battery, Gaussian process regression, Comparative analysis, Engineering, Electrical and electronic engineering

## Abstract

Accurately estimating Battery State of Charge (SOC) is essential for safe and optimal electric vehicle operation. This paper presents a comparative assessment of multiple machine learning regression algorithms including Support Vector Machine, Neural Network, Ensemble Method, and Gaussian Process Regression for modelling the complex relationship between real-time driving data and battery SOC. The models are trained and tested on extensive field data collected from diverse drivers across varying conditions. Statistical performance metrics evaluate the SOC prediction accuracy on the test set. Gaussian process regression demonstrates superior precision surpassing the other techniques with the lowest errors. Case studies analyse model competence in mimicking actual battery charge/discharge characteristics responding to changing drivers, temperatures, and drive cycles. The research provides a reliable data-driven framework leveraging advanced analytics for precise real-time SOC monitoring to enhance battery management.

## Introduction

Due to the severe consequences of climate change, environmentally favourable initiatives to reduce greenhouse gas emissions from fossil fuel combustion are urgently needed^1^. In 2023, transportation accounted for about 24% of global CO_2_ emissions from fuel combustion^2^. Electric vehicles (EVs), which produce fewer emissions than conventional petrol or diesel vehicles^3^, are seen as a potential solution. Given the urgency of climate change, precise State of Charge (SOC) estimation becomes crucial for optimizing EV performance and maximizing their environmental benefits. The choice of battery technology critically impacts EV performance. Lead-acid, nickel-metal hydride (NiMH), and lithium-ion batteries have been considered for EV applications^4^. Lithium-ion batteries (LIB) offer higher energy density, lower self-discharge, higher cell voltage, and longer cycle life compared to the other batteries^5^. LIBs also possess low weight, wide temperature range, no memory effect, and eco-friendliness^6^. The Characteristic comparison of various lithium-ion batteries is shown in Table 1. Though LIB has many advantage compared to other batteries, still battery safety, reliability, efficiency, and sustainability remain critical challenges for EVs^7^. LIBs face significant challenges, including potential failures leading to thermal runaway and accidents, raising safety concerns^8–10^.

**Table 1:** Comparison of Lead-Acid, Nickel-based, and Lithium-ion Batteries for EV Applications^11,12^.

Parameter	Lead-acid	Nickel–cadmium	Nickel-metal hydride	Lithium-ion
Energy density (Wh/L)	30–50	40–60	60–120	250–730
Energy density (Wh/kg)	25–35	45–80	60–110	100–265
Self-discharge rate	High, 5% per month	Medium, 15–20% per month	Low, 30% per year	Very low, < 2% per month
No. of charge/discharge cycles	200–300 cycles	1500 cycles	500–1000 cycles	1000 + cycles
Cost per kWh	Low	Medium	High	Medium to high
Safety	Poor due to corrosion, spillage	Poor due to high toxicity	Better	Excellent due to advanced BMS
Charge acceptance	Poor	Fair	Good	Excellent
Low temp. performance	Poor, severely degraded	Poor	Good	Excellent maintains capacity
Cell voltage stability	Poor, large variation	Poor	Better	Excellent, stable voltage

**Figure 1:**
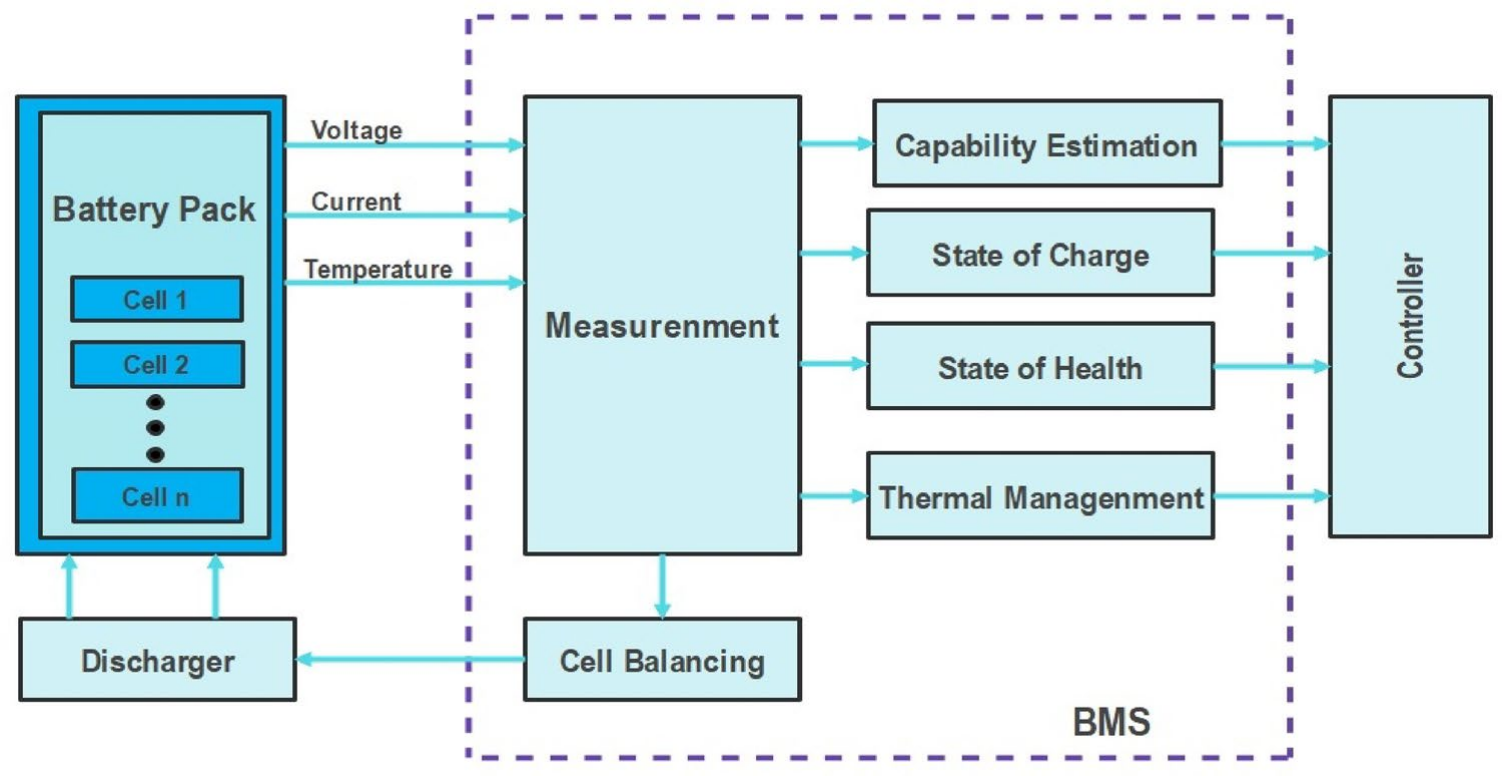
Architecture of BMS.

In order to secure safe and reliable operation of batteries, the battery management system (BMS) is highly predominant. BMS is defined as the electronic circuit that includes both hardware and software systems that collects the battery data, monitor its operation and controls its functions. The major functions of BMS includes, prediction of SOH, SOC, SOE, SOP, thermal management, and cell balancing. The structure of BMS is shown in Fig. 1. Among all the functions, SOC prediction plays the crucial role. SOC of the battery is defined as the amount of charge present in the battery to its nominal capacity under the same operating environment. Determining battery SOC is critical for EV battery management systems to ensure safe, reliable, and efficient operation^13^. However, precisely and adaptively estimating SOC under dynamic driving conditions remains an enormous challenge^14^. Better State of Charge (SOC) estimates of Lithium Ion Batteries (LIBs) used in Electric Vehicles (EVs) might minimise greenhouse gas emissions in several ways:

### Enhanced range and efficiency

Accurate battery charge information enables drivers to optimise driving patterns and avoid unexpected power outages. This can minimise EV drivers’ range anxiety and encourage more people to switch to EVs.

### Reduced regenerative braking

This technique transforms kinetic energy from the car’s motion into electrical energy for battery recharge. At high speeds, excessive regenerative braking is inefficient. By knowing the SOC better, the regenerative braking system may be optimised to capture energy efficiently without squandering it.

### Enhanced battery life

Accurate SOC estimation enables improved battery management, reducing deep discharges and high temperatures. This can lengthen battery life, decreasing replacements and the environmental effect of battery production and disposal.

### Optimized charging

Accurate SOC enables optimised charging strategies. Avoiding unnecessary battery topping off can increase battery health and efficiency. EV charging during off-peak hours, when the electricity grid may have more renewable energy, can further lower its carbon footprint.

Numerous methods has been proposed by the researchers for the prediction of SOC of LIB^15^. Classical SOC determination techniques include Coulomb Counting and Open Circuit Voltage method. Coulomb counting simply integrates cell current over time to calculate SOC based on known initial state and rated capacity^16^. However, error accumulation from current measurement and capacity deviation degrades its fidelity over operating life^17^. The OCV methods are used in predicting SOC of batteries used in laboratories only since they requirement of high battery resting time. Later model based approaches (Electric Circuit Model, Electro chemical model) observer (Sliding Mode Observer, Luenberger Observer, Non liner Observer) and filter (Kalman, particle, H infinity) based approaches has been proposed. The major drawback of model based estimation is they are affected by the aging of the battery and environmental conditions. More complex equivalent circuit modelling aims to capture cell impedance changes but still demonstrates simplistic assumptions of linear or simplified nonlinear battery behaviors, unable to mimic intricacies under varying loads, temperatures, and lifetimes^18,19^. The primary limitation of filter and observer based methods is its reliance on hardware setup and the impact of parameter variations on the accuracy of similar models. The estimation technique and battery model are unable to achieve sufficient accuracy in SOC estimation due to non-linearity, inaccurate modelling, environmental effects, and ageing factors. Several academics have suggested utilising machine learning to predict SOC in order to address these problems. Data-driven solutions that extract insights purely from sensor measurements have shown vast promise in transportation electrification owing to the proliferation of automotive sensors providing substantial telemetry data related to cell voltages, currents, and temperatures across drive cycles^20^. By directly modelling machine learning systems using input/output data, their dependence on the battery model is reduced, allowing them to be applied to any battery regardless of its technology. Recent research has actively explored more sophisticated machine learning techniques leveraging their powerful function approximation capabilities to accurately reconstruct the implicit relationship between multivariate time-series driving signals and battery internal states like SOC by mining data correlations^21^. Unlike physics-based models, machine learning algorithms automatically learn the complex electrochemical mappings from data, eliminating difficult-to-measure or unidentified cell dynamics that introduce modelling deficiencies^22^. The comparison of various SOC estimation methods using data-driven methods is shown in Table 2.

**Table 2:** The comparison of the literature review.

[Refs.]	Main algorithm used	Input parameters	Dataset used	Performance metrics
^23^	Self-supervised transformer model (Deep learning)	Voltage, current, temperature	LG 18650 cell data from various drive cycles and temps	RMSE, MAE
^24^	Machine learning (SVM, GPR, Ensemble, NN)	Current, voltage, capacity, temperature	Panasonic 18650PF dataset	Not mentioned
^25^	GRU	Voltage, current, temperature	Panasonic 18650PF Dataset and Samsung 18650-20R Dataset	MAX, MAE
^26^	BGRU with TPE optimization	Not mentioned	Custom dataset from battery test bench	RMSE, MAE
^27^	LSTM + Bayesian optimization	Voltage, current, temperature	LG 18650 HG2 dataset with drive cycles	RMSE, MAE
^28^	ELM + GSA	Current, voltage, temperature	BJDST, US06 cycles	RMSE, MAE, MSE, MAPE
^29^	Improved ELM + SSA/SCA-SSA	Current, voltage, temperature	Custom dataset from energy storage device	MAE, MAPE
^30^	ELM + GSA	Current, voltage, temperature	BJDST, US06 cycles	RMSE, MSE, MAE, MAPE
^31^	Support vector regression	Not mentioned explicitly	Battery dataset with drive cycles	RMSE, Max error
^32^	Artificial neural network	Voltage, current, temperature, avg. voltage, avg. current	In-house BMS dataset	MSE, MAE, Max error
**Proposed methodology**	**ANN, SVR and GPN with BO hyperparameter selection**	**Humidity, atmospheric temperature, battery V, I, T FET and motor temperature**	**Custom dataset from EV**	**RMSE, MSE and MAE**

Significant values are in bold.

Support vector machines are among the most widely investigated machine learning strategies for SOC estimation given their proficiency in resolving nonlinear patterns and noise tolerance stemming from kernel-based function transformation^33^. Various neural network architectures have also been evaluated given their theoretical potential to approximate virtually any nonlinear system arbitrarily well^34^. Their layered hierarchical feature transformations provide representational power to capture subtle nuances and adapt to evolving cell characteristics. Comparatively, Gaussian process regression offers a probabilistic Bayesian perspective for uncertainty quantification along with SOC prediction through kernel-based covariance modelling to inform reliability^35^.

While data-driven approaches have demonstrated promising outcomes in enhancing SOC accuracy, they also possess several limitations. The SOC prediction effectiveness is determined by a number of data-driven factors, such as the training procedure, input selection, and tuning of hyperparameters. The computational complexity of data-driven algorithms can be decreased, as well as the problems of data under fitting and overfitting, by carefully choosing the hyperparameters^36^. The hyperparameters and functions that data-driven approaches are often trained with are typically chosen through a time-consuming and inefficient trial-and-error procedure. Consequently, a great deal of investigation is needed to find a workable framework and optimise the hyperparameters of data-driven algorithms to achieve accurate SOC estimates. While the hybrid models have shown encouraging results, there is still room for development as these models have only taken into account a portion of the variables that could influence predictions and have not taken environmental factors into account. In^37^, the effect of auxiliary loads like heating and air conditioning should have been taken into account. Numerous studies^38^ discovered that an electric vehicle’s energy consumption is influenced by various factors, including road condition, traffic and environmental condition. Therefore, for a correct calculation of SOC, the impact of all these elements must be considered.

The Key contribution of the proposed article is:

- Estimate SOC of LIB using various ML regression models and compare their performance by considering EV motor characteristics, vehicle speed and environmental conditions as the input parameters along with battery parameters.
- Selection of highly correlated input parameters by applying MRMR algorithm based feature selection
- Optimal hyperparameter selection using Bayesian optimisation technique.

The research will provide significant insights into designing next-generation intelligent EV battery monitoring systems leveraging advanced data science and real-time sensor integration.

## Significance of machine learning algorithms

The accurate determination of battery SOC is vital for ensuring the safe, reliable and optimal performance of lithium-ion batteries in EV applications^21^. However, precisely estimating SOC is enormously challenging owing to the intricate nonlinear dynamics governing battery charge and discharge behaviours in response to real-world operating conditions. Factors such as cell chemistry, capacity fading, temperature fluctuations, load profiles and aging effects interact in complex ways to impact SOC evolution. Conventional model-based techniques like ampere-hour counting and open circuit voltage mapping demonstrate limited accuracy due to simplified assumptions and lack of adaptability^39^. Data-driven ML algorithms present a sophisticated solution through their unparalleled ability to uncover subtle patterns in multivariate time-series data^40^. By recognizing the implicit correlations between sensor measurements of current, voltage, temperature and SOC, they can effectively learn the battery’s internal charge dynamics^41^. The key reasons ML methods are hugely significant for realizing accurate SOC prediction are superior function approximation capabilities, adaptability to changing conditions, high noise tolerance, modelling complexity handling, and real-time prediction.

### Support vector machines

**Figure 2:**
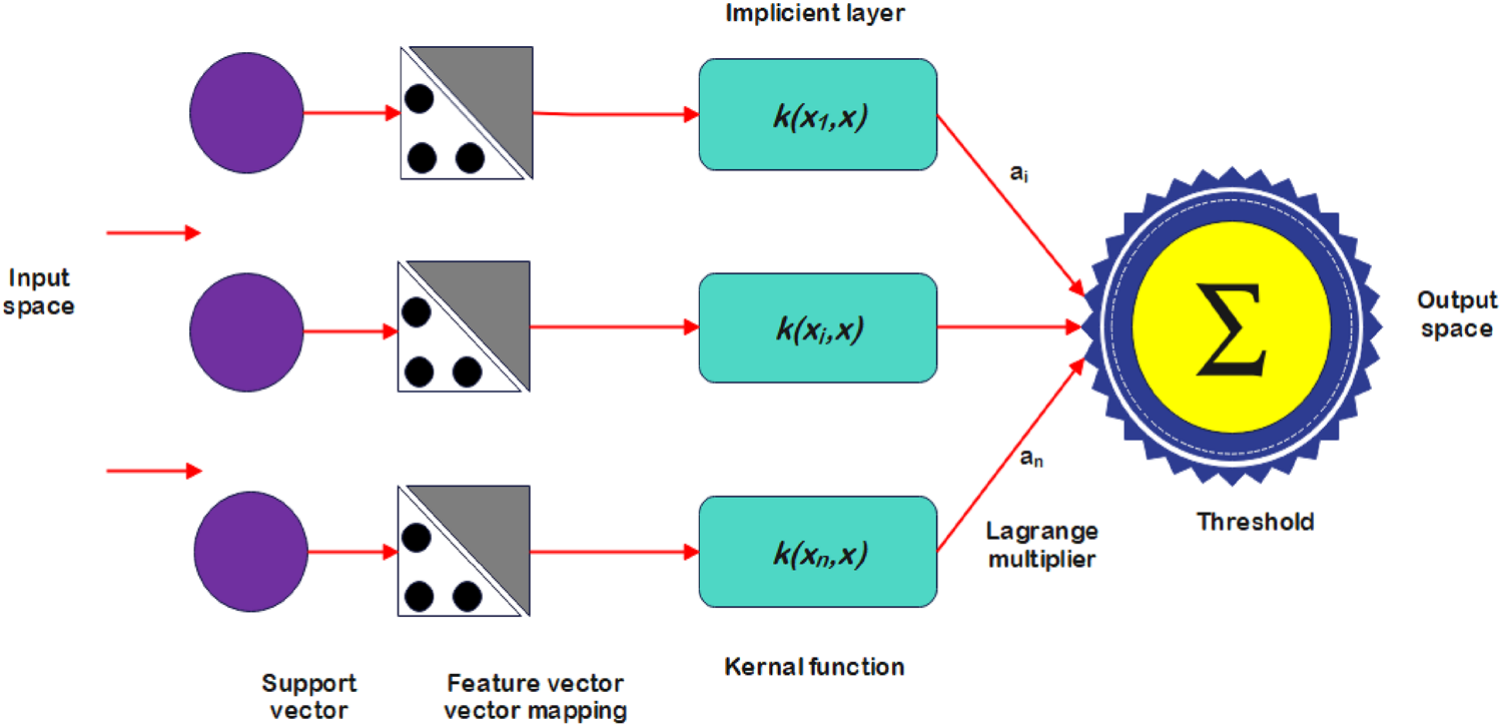
General architecture of SVM.

SVMs are widely utilized in classification and regression problems owing to their proficiency in nonlinear function approximation, high dimensional feature space handling and global optima convergence^41^. At the core, SVMs employ kernel functions to implicitly transform input data into elevated dimensional spaces where linear decision boundaries can separate classes or fit nonlinear trends. The key concept underpinning the working of SVMs is the kernel trick efficiently computing dot products between inputs in the transformed space using a kernel function without needing to explicitly calculate the high dimensional mapping. Some commonly used SVM kernels include polynomial, radial basis function, and sigmoid kernels. The basic architecture of SVM is given in Fig. 2 and its kernel is listed in Table 3.

**Table 3:** SVM kernel equations^42^.

Variant	Kernel equation
Linear SVM	$k\left( {x,x^{\prime}} \right) = x^{T} x^{\prime}$
Quadratic SVM	$k\left( {x,x^{\prime}} \right) = \left( {x^{T} x^{\prime} + c} \right)^{2}$
Cubic SVM	$k\left( {x,x^{\prime}} \right) = \left( {x^{T} x^{\prime} + c} \right)^{3}$
Fine Gaussian SVM	$k\left( {x,x^{\prime}} \right) = {\text{exp}}\left( { - \frac{{\left\| {x - x^{\prime}} \right\|^{2} }}{{2\sigma_{f}^{2} }}} \right)$
Medium Gaussian SVM	$k\left( {x,x^{\prime}} \right) = {\text{exp}}\left( { - \frac{{\left\| {x - x^{\prime}} \right\|^{2} }}{{2\sigma_{m}^{2} }}} \right)$
Coarse Gaussian SVM	$k\left( {x,x^{\prime}} \right) = {\text{exp}}\left( { - \frac{{\left\| {x - x^{\prime}} \right\|^{2} }}{{2\sigma_{c}^{2} }}} \right)$

### Artificial neural networks

**Figure 3:**
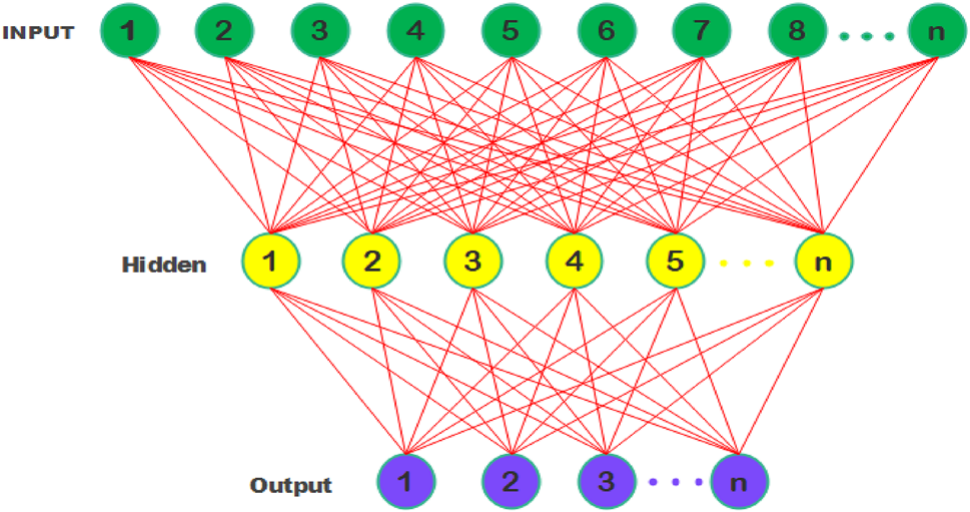
Neural network structure.

Artificial neural networks are computational models inspired by the biological neural networks in human brains. They consist of an interconnected web of artificial neurons organized in layers that transmit signals via weighted connections analogous to biological synapses^43^. The hierarchical multilayer network architecture provides the representational power to approximate virtually any nonlinear function with arbitrarily high accuracy given sufficient hidden neurons^44^. At the heart of a neural network’s computational logic is its ability to learn from experience by tuning its synaptic weights using the backpropagation algorithm minimizing the error in network’s outputs by recursively propagating derivatives towards earlier layers^45^. The universal approximation theorem establishes that even simple feedforward networks with a single layer can represent any continuous function, while deeper networks learn highly nonlinear relationships more efficiently^46^. The basic architecture of SVM is given in Fig. 3 and its kernel is listed in Table 4.

**Table 4:** Kernel detail for ANN^47^.

Variant	Architecture
Narrow Neural Network	Shallow architecture with fewer neurons or layers
Medium Neural Network	Moderate depth and width
Wide Neural Network	More neurons per layer or more layers
Bilayered Neural Network	Two layers with specified neurons
Trilayered Neural Network	Three layers with specified neurons

### Gaussian process regression

**Figure 4:**
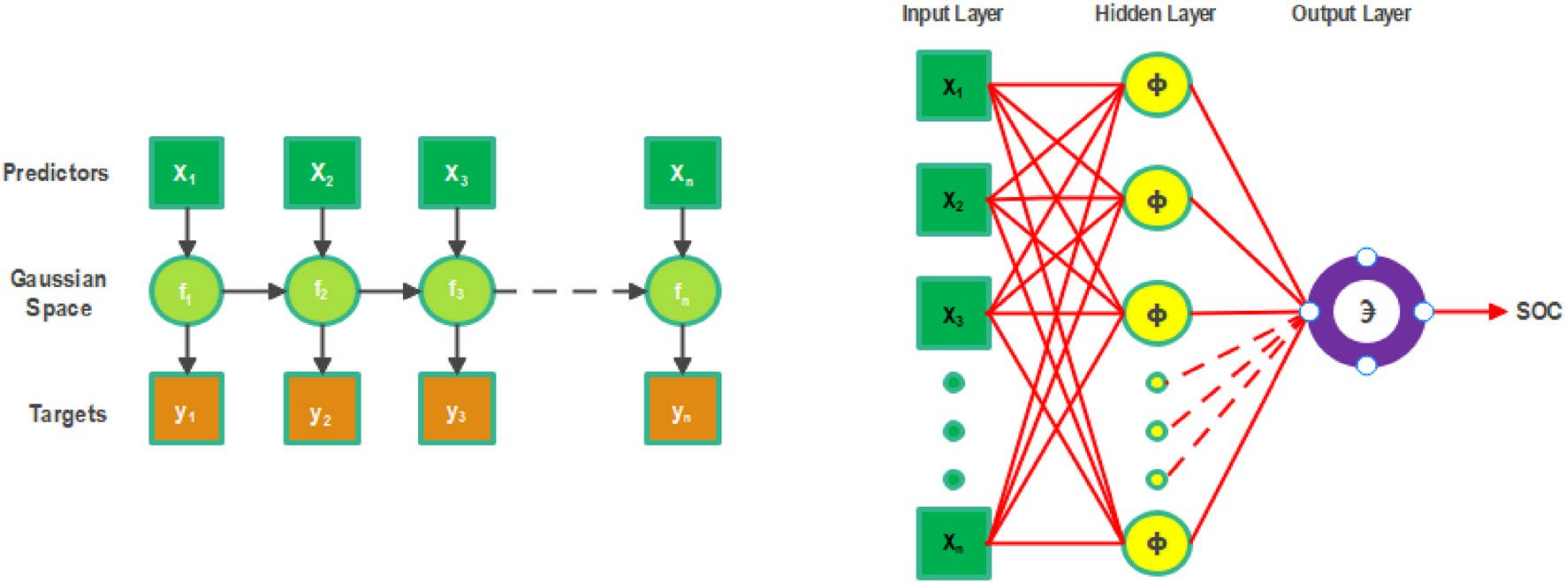
Gaussian process regression model.

While neural networks formulate function approximation as optimization over parametric model families, Gaussian processes provide a Bayesian nonparametric perspective^48^. A Gaussian process defines a distribution over possible functions consistent with the training data instead of explicitly learning network weights. It is fully specified by its mean and covariance functions, with the latter quantifying the correlation structure governing how function values co-vary across input points^49^. Gaussian process regression considers function evaluation as a stochastic process, with observations getting projected to an associated normal distribution characterized by uncertainty^40^. The basic architecture of SVM is given in Fig. 4 and its kernel is listed in Table 5.

**Table 5:** GPR kernel equations^50^.

Variant	Kernel equation
Squared Exponential GPR	$k\left( {x,x^{\prime}} \right) = \sigma^{2} {\text{exp}}\left( { - \frac{{\left\| {x - x^{2} } \right\|^{2} }}{{2l^{2} }}} \right)$
Matern 5/2 GPR	$k\left( {x,x^{\prime}} \right) = \sigma^{2} \left( {1 + \frac{{\sqrt 5 \left\| {x - x^{\prime}} \right\|}}{l} + \frac{{5\left\| {x - x^{\prime}} \right\|^{2} }}{{3l^{2} }}} \right){\text{exp}}\left( { - \frac{{\sqrt 5 \left. {\left| {x - x^{\prime}} \right.} \right\|}}{l}} \right)$
Exponential GPR	$k\left( {x,x^{\prime}} \right) = \sigma^{2} {\text{exp}}\left( { - \frac{{\left\| {x - x^{\prime}} \right\|}}{l}} \right)$
Rational Quadratic GPR	$k\left( {x,x^{\prime}} \right) = \sigma^{2} \left( {1 + \frac{{\left\| {x - x^{\prime}} \right\|^{2} }}{{2\alpha l^{2} }}} \right)^{ - \alpha }$

## The proposed SOC estimation methodology

**Figure 5:**
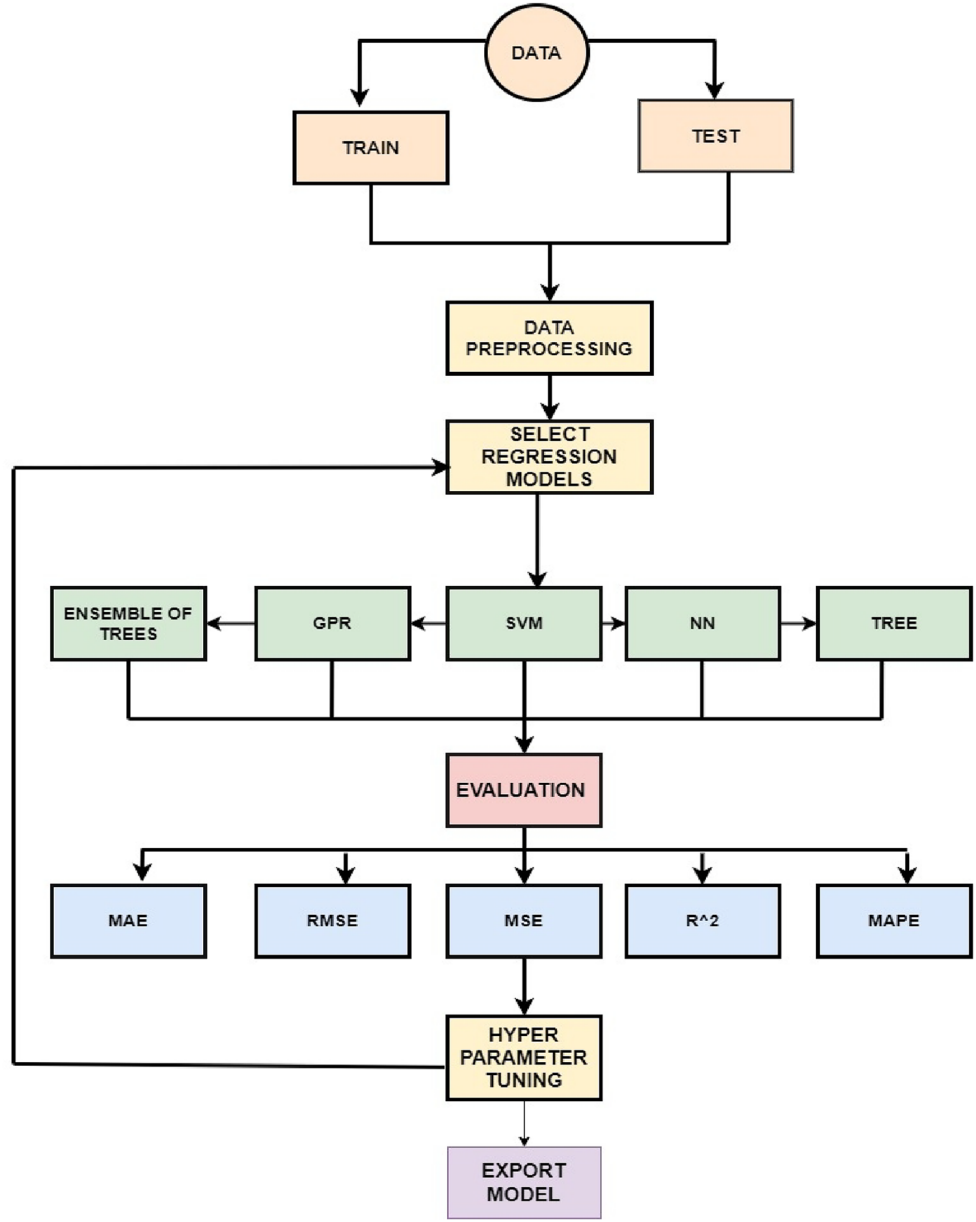
Flowchart of the proposed SOC estimation methodology.

The workflow of SOC estimation of batteries using ML algorithms is given in the Fig. 5. The methodology for estimating SOC follows a systematic flowchart consisting of key steps including data separation, data pre-processing, model selection, evaluation, and hyperparameter tuning. This allows for an organized approach to developing and optimizing an accurate SOC estimation model.

### Data collection and preprocessing

The utilization of the DAQ module facilitated the extraction of real-time data from a battery-EV across diverse scenarios. The driver of the vehicle was altered on a daily basis during the data collecting period, leading to fluctuations in both age and weight. It was believed that there would be a variation in driving style corresponding to the age of the driver. Furthermore, a data collection process was conducted for a duration of 30 consecutive days in order to facilitate the observation and recording of externally temperature variations. The purpose of this experiment was to investigate the potential impact of varying temperature conditions on the extracted data. Additionally, the collection of data also accounted for traffic circumstances and road conditions, as these were deemed to potentially influence the SOC of the vehicle. As a result, a complete dataset was acquired, encompassing over 30 parameters such as battery voltage, battery current, temperature, and motor speed. Various pre-processing steps were conducted to prepare the data for subsequent analysis. The process included the rounding of data, removal of duplicates, and adjustment of variable values to enhance accessibility and user-friendliness. Furthermore, the process of feature scaling was implemented on many variables, resulting in their values being transformed to a standardized range of 0–1, while simultaneously maintaining the original parameter value. The objective of feature scaling was to enhance the training process of the algorithmic model. Finally, in order to conduct training and testing on the data, the 60/40 principle was adhered to. This involved allocating 60% of the data for training the model, while the remaining 40% was used for testing purposes.

### Feature selection

**Figure 6:**
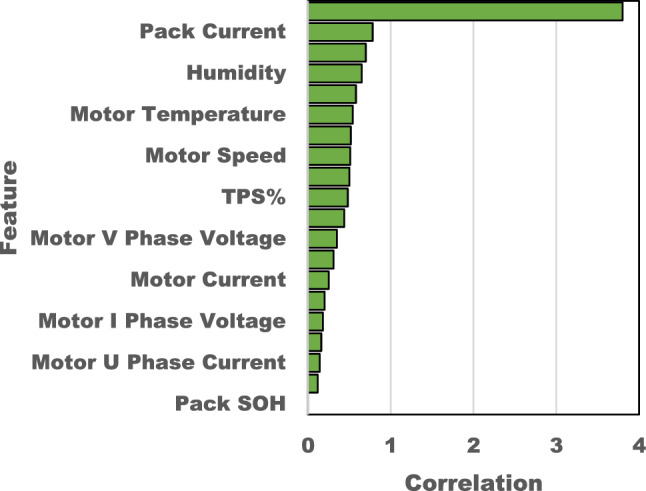
Feature scaling of input parameters.

**Figure 7:**
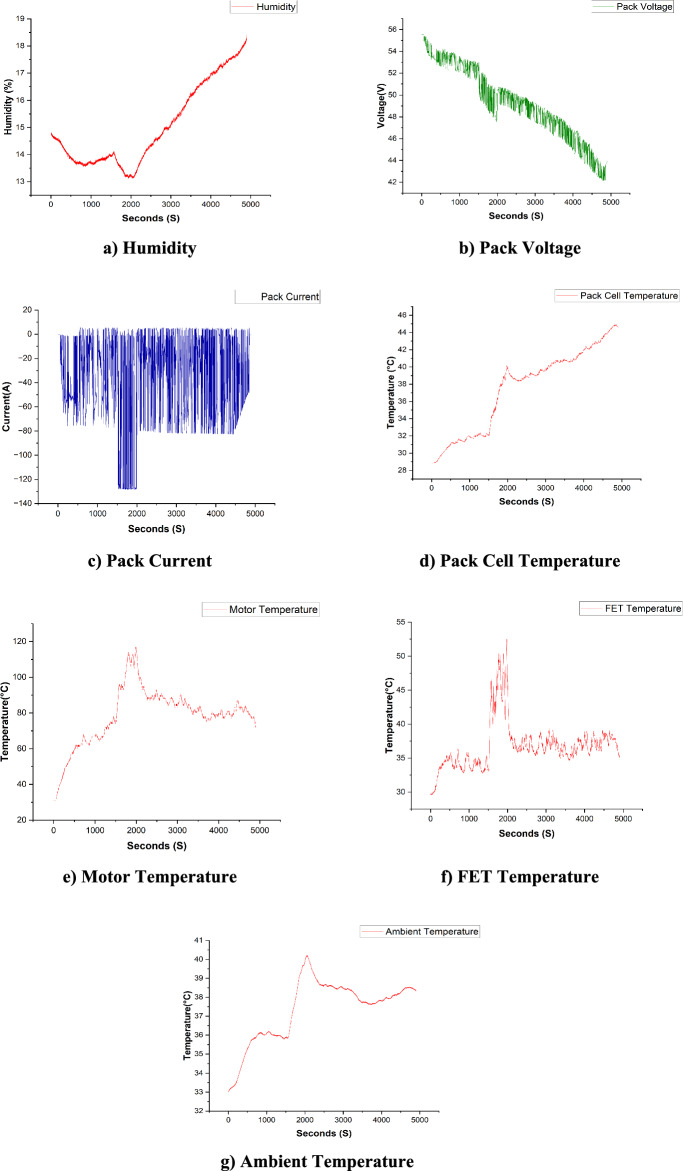
Input parameters. (**a**) Humidity. (**b**) Pack voltage. (**c**) Pack current. (**d**) Pack cell temperature. (**e**) Motor temperature. (**f**) FET temperature. (**g**) Ambient temperature.

The procedure of feature selection is conducted using the MATLAB software. The utilization of feature selection approaches in data analysis is of great significance, especially due to a convergence of compelling factors. First and foremost, the process of feature selection plays a significant role in improving the interpretability of a model^51^. By identifying and retaining just the most relevant input variables, feature selection simplifies the underlying model for estimating SOC. The process of simplification serves two important purposes: enhancing comprehension of the connections between input characteristics and SOC, and improving the computational efficiency of the model^52^. Additionally, the utilization of feature selection techniques assists in addressing the challenge of the curse of dimensionality, a phenomenon that is especially pertinent in the analysis of battery data. This is due to the fact that battery datasets frequently consist of a large number of input factors. Feature selection mitigates overfitting, enhances the generalizability of models, and guarantees reliable state-of-charge (SOC) predictions in the presence of diverse operating conditions through the reduction of data dimensionality. Moreover, it improves the model’s flexibility, making it more resistant to variations in the battery system’s arrangement or chemical composition, which is a vital aspect in accurately estimating the SOC in real-time for different types of batteries. In addition, the process of feature selection contributes to the optimization of resources by minimizing computing demands and memory requirements. This aligns with the efficiency requirements of real-time applications. Finally, it improves the dependability of the model by reducing the impact of extraneous or repetitive characteristics, hence strengthening the precision of SOC estimations. Figure 6 is plotted with all accessible datasets that can be retrieved in real time using DAQ, and it ranks itself based on its strong association with SOC. From the same figure we were able to conclude that top eight factors named as pack cell temperature, pack current, FET temperature, humidity, ambient temperature, motor temperature, pack voltage and motor speed are associated in in predicting SOC. The input parameters selected for simulation are plotted in Fig. 7.

### Model selection

Since the objective of this paper is to predict and estimate the SOC using machine learning techniques, the regression models available in the field of machine learning have been utilized. The reason behind using regression models lies in their ability to effectively capture the relationship between variables and make accurate predictions. Various regression analysis techniques are employed when there exists a linear or non-linear association between the target and independent variables, and the target variable comprises continuous values. Regression analysis is widely recognized as the predominant methodology employed in machine learning for addressing regression problems through the application of data modelling techniques. The process includes the identification of the optimal fit line, which is a line that intersects with all data points in a manner that minimizes the distance between the line and each individual data point. Based on the present study, a number of machine learning models were chosen, including SVM, NN, Ensemble of Trees, and Gaussian Process Regression (GPR). Additionally, to mitigate the issue of overfitting, the extended Kalman filter (EKF) was employed. The EKF method possesses the capability to adaptively modify the estimation procedure in order to reduce the issue of overfitting, the utilization of a system model in the estimate process is a fundamental characteristic of the EKF.

### Hyper parameter optimization

Choosing proper model hyperparameters goes about as a difficult undertaking in AI calculations. The model’s performance can be affected by fine-tuning its hyperparameters. A few examples of hyperparameters for profound learning techniques include the number of units in each layer, the number of layers, the activation function in each layer, the optimizer, the learning rate, and so on. More model parameters require a more extensive search hyperspace. Exhaustive techniques, such as grid search (GS) or random search (RS), are computationally costly and tedious because of the huge dimensionality of hyperspace. Thus, probabilistic models, including the Bayesian Optimization algorithm, have been investigated to tune high-cost evaluation models. The hyperparameters of the ML algorithms model displayed here were chosen utilizing a Bayesian Optimization method. A surrogate model of the goal function is created in Bayesian optimization, and only the hyperparameters that demonstrate the greatest promise based on the outcomes of previous trials are evaluated.

#### Support vector machine (SVM)

The SVM algorithm has hyperparameters that control the kernel type, the kernel coefficients, and the regularization parameter. These parameters affect the decision boundary learned by the SVM. Kernel parameters control the shape of the decision boundary, while C controls the trade-off between misclassification and simplicity of the boundary. The details of SVM hyperparameter range and its optimised hyperparameter is given in Table 6.

**Table 6:** Hyper parameter details for SVM.

Hyperparameter	Hyperparameter range	Optimized hyperparameter
Kernel function	Linear, Quadratic, Cubic, Fine Gaussian, Medium Gaussian, Coarse Gaussian	RBF
Gamma (kernel coefficient)	0.01–10	0.5
Cost (regularization)	1–100	10
Epsilon	0.001–0.1	0.01

#### Gaussian process regression (GPR)

Key hyperparameters of Gaussian process regression include the kernel function and its parameters (analogous to the SVM), along with parameters that determine the noise level of the data and optimize the trade-off between data fit and model complexity. Common kernels include squared exponential, Matern, and rational quadratic. Kernels have parameters like length scales that control how quickly the correlations decay with distance. The details of GPR hyperparameter range and its optimised hyperparameter is given in Table 7.

**Table 7:** Hyper parameter details for GPR.

Hyperparameter	Hyperparameter range	Optimized hyperparameter
Kernel function	Squared Exponential, Matern 5/2, Exponential, Rational Quadratic,	Matern 5/2
Length scale	0.1–10	1.3
Signal variance	0.1–5	1.7
Noise level	0.01–1	0.05

#### Neural network (NN)

Neural networks have numerous hyperparameters including the number and width of hidden layers, activation functions, weight initialization schemes, batch size, number of training epochs, and optimizers. Most modern NNs use adaptive optimization methods like Adam or RMSprop which have additional hyperparameters like learning rate and scheduling. Early stopping is also commonly used, adding another hyperparameter for patience. The hyperparameter values for the machine learning models used here were selected using Bayesian optimization. Bayesian optimization builds a probabilistic surrogate model for the objective and samples hyperparameters that appear most promising based on previous evaluation results. This allows efficient tuning of costly models with many hyperparameters. The details of ANN hyperparameter range and its optimised hyperparameter is given in Table 8.

**Table 8:** Hyperparameter details for NN.

Hyperparameter	Hyperparameter range	Optimized hyperparameter
Number of hidden layers	01–2	1
Number of neurons per layer	10–100	64
Activation function	Tanh, ReLU	Tanh
Solver	Adam, RMSprop	Adam
Learning rate	0.001–0.1	0.01

## Validation of the ML model

To comprehensively assess the effectiveness of the constructed machine learning (ML) models, a range of statistical error functions were employed. The root-mean-squared error (RMSE), mean absolute error (MAE), and the coefficient of determination (R^2) were calculated to evaluate the models’ predictive capabilities.

$$\begin{array}{c} \, {\text{R}}{\text{M}}{\text{S}}{\text{E}} \, =\sqrt{\sum_{j=1}^{N} \frac{{\left({z}_{j}-{\widehat{z}}_{j}\right)}^{2}}{N}}\\ MAE=\sum_{j=1}^{N} \frac{\left|{z}_{j}-{\widehat{z}}_{j}\right|}{N}\\ {R}^{2}=1-\frac{\sum_{j=1}^{N} {\left({\widehat{z}}_{i}-{z}_{i}\right)}^{2}}{\sum_{j=1}^{N} {\left({\widehat{z}}_{j}-{\widehat{z}}_{\text{ave }}\right)}^{2}}\end{array}$$

${z }_{j}$ Represents the predicted data, $\widehat{z}_{j}$ stands for the target datasets and the term “n” indicates the number of dataset observations.

The RMSE is a widely adopted metric that quantifies the square root of the mean squared differences between the predicted and actual values. Lower RMSE values indicate better model performance, as they signify that the predicted values are more concentrated around the line of best fit. However, it is important to note that the RMSE is sensitive to outliers, as squaring the errors amplifies the impact of larger deviations. In contrast, the MAE calculates the average magnitude of the errors without considering their direction. Unlike the RMSE, the MAE is less influenced by outliers, as it does not involve squaring the errors. Nonetheless, interpreting the MAE can be more challenging compared to the RMSE, as it lacks the same natural interpretation in terms of the data’s units. The coefficient of determination (R^2^) provides a measure of how well the predicted values from the model fit the actual data. It represents the proportion of the variance in the dependent variable that can be explained by the independent variables in the model. R^2^ values range from 0 to 1, with higher values indicating a better model fit. However, it is crucial to recognize that a high R^2^ value does not necessarily imply a good model, as it can be influenced by the model’s complexity and the presence of irrelevant variables.

## Results and discussion

### Experimental setup

**Figure 8:**
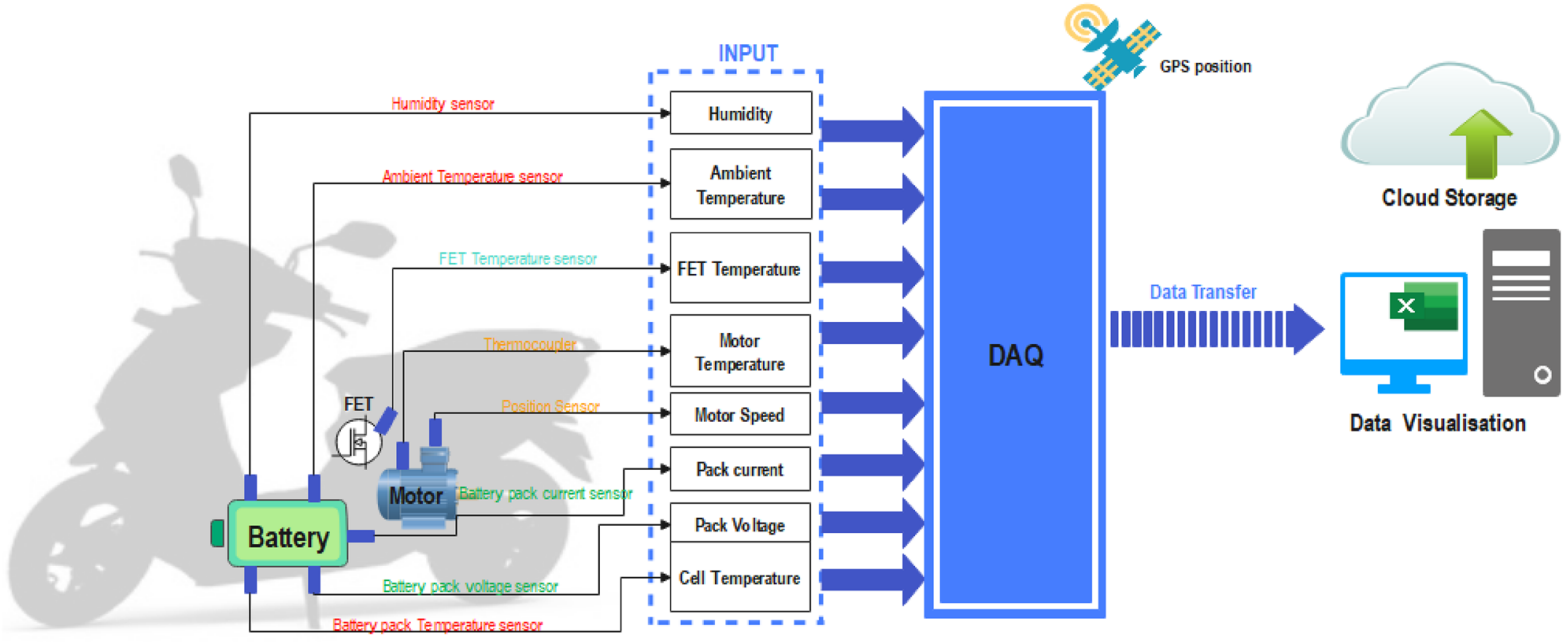
Schematic diagram of the proposed experimental setup.

In order to conduct real-time data extraction for a variety of driving cycles, it is vitally important to employ a meticulously developed experimental configuration. This configuration is suitable for gathering essential performance data throughout the operation of a vehicle. The experimental setup of the proposed work is shown in the Fig. 8. An appropriate EV, equipped with the requisite sensors, is chosen ensuring that the vehicle’s motor control system supports data output via the On-Board Diagnostics (OBD) interface. The NI is safely fitted into the designated EV, enabling the acquisition of analogue and digital input channels with superior quality. The car is equipped with supplementary sensors, including a GPS system for tracking its position, a thermocouple for monitoring engine temperature, a temperature sensor for monitoring battery temperature, FET and ambient temperature, humidity sensor to monitor humidity, and a battery voltage and current sensor.

The connection interface serves as an intermediary between the sensors and the data acquisition (DAQ) system. The utilization of a specialized data logging programme that establishes a connection with the DAQ hardware enables the acquisition of real-time data from vehicle sensors, thereby initiating the process of data storage. The DAQ system is linked to the OBD port of the vehicle in order to get essential vehicle parameters, including speed, throttle position, RPM, and other relevant data. The integration of GPS data with the DAQ system enables the precise recording of vehicle location and speed, which is of utmost importance in the generation of a driving cycle. The monitoring of data quality and sensor accuracy is an ongoing process throughout several driving cycles. The data that has been retrieved is safely stored in the CSV format, allowing for easy accessibility. The CSV format includes time stamp data that utilizes GPS information. The retrieved data undergoes essential filtration processes in order to generate a SOC value for the whole driving cycle.

### Model evaluation without hyper parameter tuning

The model training and testing was carried out in Matlab 2023a version. Several machine learning models were trained to predict an output variable from input data. The models included trees, Gaussian process regression, linear regression, support vector machines, neural networks, ensembles, and kernel methods. Each algorithm has a set of tuneable hyperparameters that control model complexity, overfitting, and other properties. The models were trained using default hyperparameters without tuning on a separate validation dataset.

#### Train results

**Figure 9:**
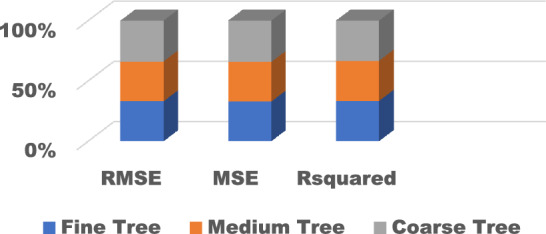
Comparison of performance indices of different kernels of tree.

**Figure 10:**
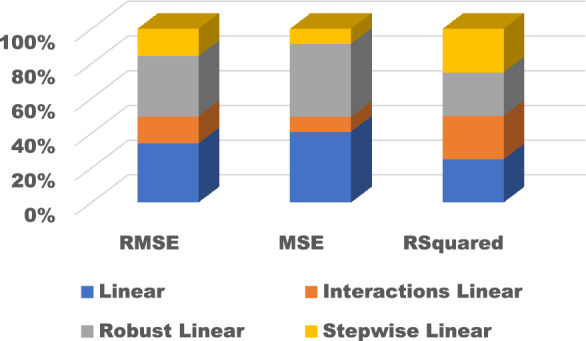
Comparison of performance indices of different kernels of linear regression.

**Figure 11:**
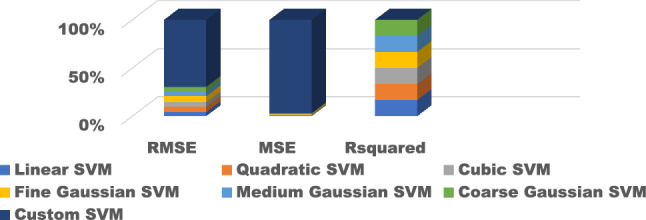
Comparison of performance indices of different kernels of SVM.

**Figure 12:**
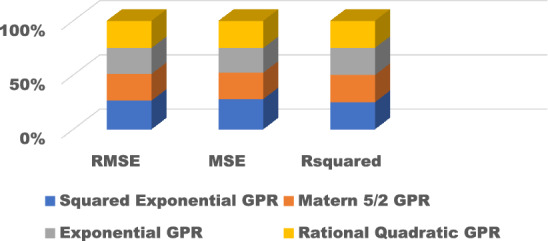
Comparison of performance indices of different kernels of GPR.

**Figure 13:**
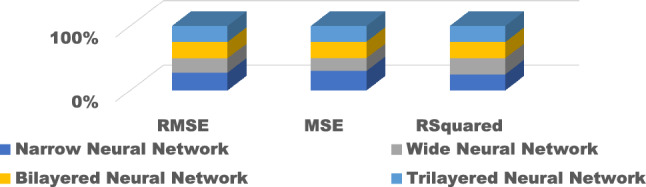
Comparison of performance indices of different layers of neural network.

The training results for the different models with hyperparameter tuning are shown in Figs. 9, 10, 11, 12 and 13. The Gaussian process regression model achieved the lowest root mean squared error (RMSE) of 0.0015 and highest R-Squared of 0.9999 during training. Other top-performing models on the validation data included the stepwise linear regression, narrow neural network, and Matern 5/2 Gaussian process regression which obtained RMSE scores below 0.003.

#### Test results

**Figure 14:**
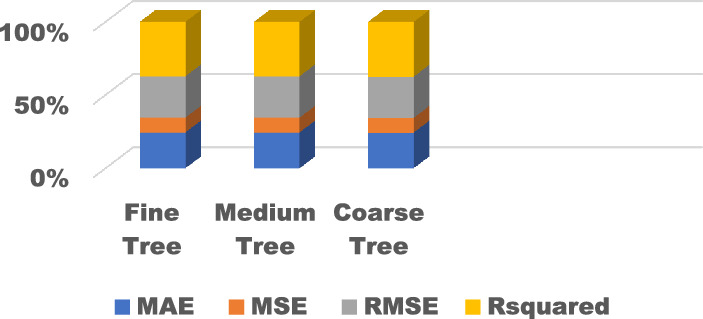
Comparison of performance indices of different kernels of tree during testing.

**Figure 15:**
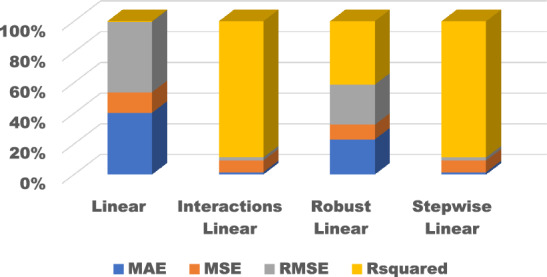
Comparison of performance indices of different kernels of linear regression during testing.

**Figure 16:**
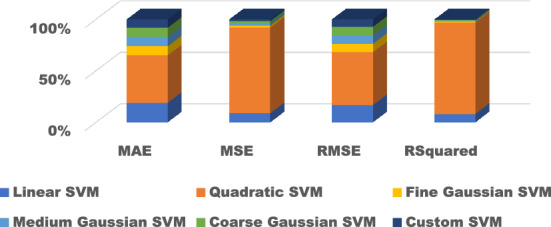
Comparison of performance indices of different kernels of SVM testing.

**Figure 17:**
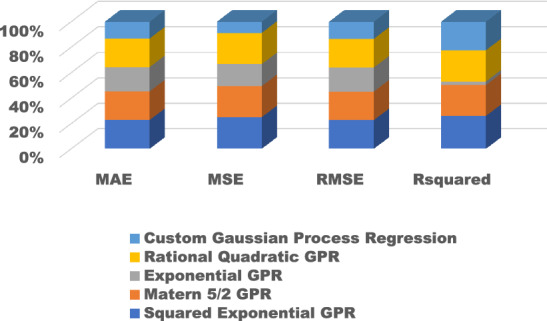
Comparison of performance indices of different kernels of GPR during testing.

**Figure 18:**
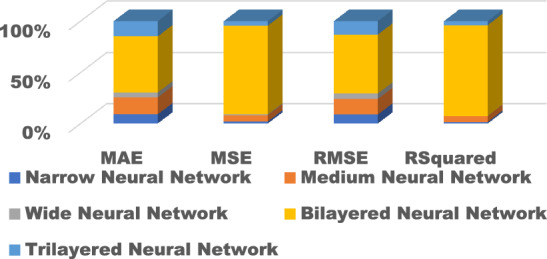
Comparison of performance indices of different layers of neural network during testing.

The test results evaluate model generalization error to new unseen data. As shown in Figs. 14, 15, 16, 17 and 18, many models exhibited overfitting with much higher error metrics on the test data versus validation. The ensemble boosted trees model performed the best on testing with the lowest mean absolute error of 0.22 and R-Squared of 0.23. Other models with reasonable test performance include the exponential Gaussian process model and wide neural network. The extreme overfitting of linear regression and SVM variants highlights the need for proper regularization and hyperparameter tuning through cross-validation.

### Model evaluation with hyper parameter

**Figure 19:**
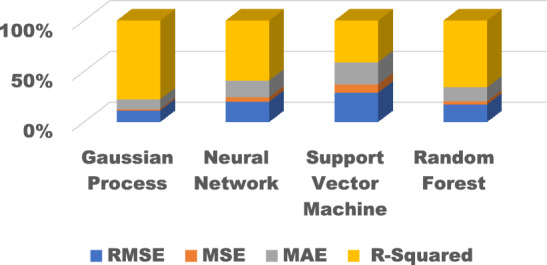
Comparison of performance indices of different ML algorithms.

To reduce overfitting exhibited by models on the test data, hyperparameter tuning was performed through cross-validation on the training set. The best cross-validation hyperparameter configuration for each model is shown in Table 9 and the overall comparison of the various ML models with hyperparameter tuning is shown in Fig. 19. By observing various ML models during testing and training with hyperparameter tuning, it is observed that the GPR has high accuracy, reduced RMSE and have high generalisation.

**Table 9:** Test performance with best hyperparameters.

Model	Best hyperparameters	RMSE	MSE	MAE	R-squared
Gaussian Process	Matern 5/2 kernel, length scale = 1.5	0.12	0.015	0.1	0.81
Neural Network	1 hidden layer, ReLU, Adam optimizer	0.21	0.044	0.17	0.61
Support Vector Machine	RBF kernel, C = 1, gamma = 0.5	0.29	0.084	0.22	0.41
Random Forest	100 trees, max depth = 5	0.19	0.036	0.15	0.72

The Gaussian process model used the Matern 5/2 kernel along with a characteristic length scale parameter that controls how quickly the correlations decay between data points. The neural network contains hyperparameters defining the topology and learning process. The support vector machine has kernel parameters along with the regularization strength C. Finally, the random forest ensemble has tuning hyperparameters for the number of decision trees and their depth. Tuning was done through random search and Bayesian optimization of ~ 20 trials per algorithm. The best configurations discovered through this tuning procedure likely improved model generalization and test performance. However, further tuning work could better optimize predictive accuracy. Overall this highlights model selection and tuning of appropriate hyperparameters as a key machine learning pipeline step before final evaluation.

### SOC estimation fidelity analysis

Further analysis was conducted using GPR model case studies across different scenarios to evaluate its fidelity in mimicking real battery behaviors:

- For a typical driver with gradual acceleration and deceleration, the GPR model accurately depicted the linear SOC discharge behaviour from 100% starting SOC down to 92% after 30 min of driving. This aligns with the stabilized discharge profile expected at high SOCs.
- At a lower starting SOC of 50%, GPR correctly captured the slightly increased battery discharge rate with SOC reducing to 35% over 30 min for the same driver and driving cycle. This reflects the nonlinear capacity effects and voltage drop generally seen at low SOCs.
- The GPR model effectively took into account the impact of diverse driving cycles on battery usage. Model testing showed much faster SOC depletion in an aggressive NYC cycle compared to an LA highway cycle for the same distance covered. This mimics increased power demand under city driving.
- GPR accurately incorporated driver behaviour effects with a calm driver discharging the battery slower compared to an aggressive driver over the identical route and distance travelled. This matches expected higher current draws with harsh acceleration/braking.
- The model reliably predicted higher battery discharge rates at elevated temperatures above 35 °C versus slower discharge at lower temperatures below 15 °C, replicating increased internal resistance losses at high temperatures.
- For a 20 mile trip, the projected ending SOCs were 91%, 72%, 65% for starting SOCs of 100%, 75% and 50% respectively. This demonstrates GPR’s competence in relating starting SOC and distance travelled to final SOC after a drive cycle.

The case study analysis highlights GPR’s excellence in capturing the intricate charge and discharge relationships of batteries in response to changing real-world conditions. The findings showcase GPR’s high model fidelity for accurately mimicking diverse scenarios reflecting actual EV operation.

## Conclusion

Accurate estimation of battery SOC is critical for effective battery management and safe operation of EVs. This study presented a comparative analysis of multiple machine learning regression techniques for modelling the complex nonlinear relationship between real-time driving data and battery SOC. The models will be improved with the incorporation of the Minimum Redundancy Maximum Relevance (MRMR) technique for feature selection and Bayesian optimisation for hyperparameter tuning. In order to improve estimation accuracy, the input parameters include battery voltage, current, environmental condition like humidity and ambient temperature and along with battery temperature, motor and FET temperature in EV. Their performance was quantified using statistical measures of coefficient of determination, root mean squared error, mean absolute error, mean absolute percentage error and mean squared error. The results demonstrated the superior accuracy and robustness of Gaussian process regression compared to the other methods. It consistently achieved the lowest errors with RMSE of 0.8% and MSE of 0.6 on the test dataset. The GPR model also exhibited excellent fidelity in mimicking real battery behaviors under diverse operating conditions. The potential challenge in training this model is the requirement of high speed system processors and the collection of real time data. In the future work, these models will be applying the developed models in practical BMS and analyse its performance.

## Data Availability

The datasets generated during and/or analysed during the current study are available from the corresponding author on reasonable request.

## Abbreviations

SOC: State of charge
ML: Machine learning
SVM: Support vector machine
NN: Neural network
GPR: Gaussian process regression
RMSE: Root mean squared error
EV: Electric vehicle
SOH: State of health
MAE: Mean absolute error
MAPE: Mean absolute percentage error
R^2^: Coefficient of determination (squared)
DAQ: Data acquisition
BMS: Battery management system
EKF: Extended Kalman Filter
SOP: State of power
SOE: State of energy
